# Barley plants over-expressing the NAC transcription factor gene *HvNAC005* show stunting and delay in development combined with early senescence

**DOI:** 10.1093/jxb/erw286

**Published:** 2016-07-19

**Authors:** Michael W. Christiansen, Colette Matthewman, Dagmara Podzimska-Sroka, Charlotte O’Shea, Søren Lindemose, Niels Erik Møllegaard, Inger B. Holme, Kim Hebelstrup, Karen Skriver, Per L. Gregersen

**Affiliations:** ^1^Department of Genetics and Biotechnology, Aarhus University, Forsøgsvej 1, Slagelse DK-4200, Denmark; ^2^Department of Biology, University of Copenhagen, Ole Maaløes Vej 5, DK-Copenhagen N, Denmark; ^3^Department of Cellular and Molecular Medicin, University of Copenhagen, Blegdamsvej 3B, DK-Copenhagen N, Denmark

**Keywords:** Barley, gene regulation, *Hordeum vulgare* L., NAC transcription factors, senescence, senescence-associated genes, transgenic plants.

## Abstract

The plant-specific NAC transcription factors have attracted particular attention because of their involvement in stress responses, senescence, and nutrient remobilization. The *HvNAC005* gene of barley encodes a protein belonging to subgroup NAC-a6 of the NAC family. This study shows that *HvNAC005* is associated with developmental senescence. It was significantly up-regulated following ABA treatment, supported by ABA-responsive elements in its promoter, but it was not up-regulated during dark-induced senescence. The C-termini of proteins closely related to HvNAC005 showed overall high divergence but also contained conserved short motifs. A serine- and leucine-containing central motif was essential for transcriptional activity of the HvNAC005 C-terminus in yeast. Over-expression of *HvNAC005* in barley resulted in a strong phenotype with delayed development combined with precocious senescence. The over-expressing plants showed up-regulation of genes involved with secondary metabolism, hormone metabolism, stress, signalling, development, and transport. Up-regulation of senescence markers and hormone metabolism and signalling genes supports a role of HvNAC005 in the cross field of different hormone and signalling pathways. Binding of HvNAC005 to promoter sequences of putative target genes containing the T[G/A]CGT core motif was shown by direct protein–DNA interactions of HvNAC005 with promoters for two of the up-regulated genes. In conclusion, HvNAC005 was shown to be a strong positive regulator of senescence and so is an obvious target for the fine-tuning of gene expression in future attempts to improve nutrient remobilization related to the senescence process in barley.

## Highlight

HvNAC005 was shown to be a strong positive regulator of senescence, involved in regulation in the cross field of different hormone and signalling pathways controlling developmental senescence in barley.

## Introduction

Leaf senescence, the final stage of development, is a complex, finely tuned process. Following the onset of senescence, there is a reduction in carbon assimilation due to the breakdown of the photosynthetic machinery for the remobilization of nutrients to the developing seeds. This makes senescence a limiting step for crop yields and its regulation a good target for crop improvements and for efficient nutrient remobilization. Substantial changes in gene expression accompany the onset of senescence with, for example, photosynthesis-related genes being down-regulated and senescence-associated genes (SAGs) being up-regulated (Guo *et al.*, 2004; Buchanan-Wollaston *et al.*, 2005). Microarray analyses of natural or induced senescence in barley have indicated similar patterns in barley (Parrott *et al.*, 2007; Christiansen and Gregersen, 2014; Hollmann *et al.*, 2014).

Several transcription factor families have been shown to be involved in the regulation of senescence, including NAC, WRKY, C2H2 type zinc finger, AP2/EREBP, MYB, HB, and bZIP proteins (Guo *et al.*, 2004), and hundreds of genes belonging to these families are differentially regulated during senescence. The plant-specific NAC transcription factors have attracted particular attention because of their involvement in both biotic and abiotic stress responses and in senescence regulation (for reviews see Olsen *et al.*, 2005*a*; Nakashima *et al.*, 2012; Puranik *et al.*, 2012; Jensen *et al.*, 2014; Podzimska-Sroka *et al.*, 2015).

The large NAC gene family has 117 members in Arabidopsis, 151 in rice, and 152 in both soybean and tobacco (Ooka *et al.*, 2003; Rushton *et al.*, 2008; Nuruzzaman *et al.*, 2010; Le *et al.*, 2011). Over-expression of *AtNAP*, *ORE1*, *ORS1*, *ANAC016*, and *ATAF1* resulted in precocious senescence, whereas repressing the function of these genes resulted in delayed senescence, suggesting that they function as non-redundant positive regulators of senescence in Arabidopsis (Guo and Gan, 2006; Kim *et al.*, 2009, 2013; Balazadeh *et al.*, 2010, 2011; Garapati *et al.*, 2015). Negative regulators of leaf senescence have also been identified among the NAC genes with over-expression of both Arabidopsis *JUB1* and *VNI2* delaying senescence (Wu *et al.*, 2012; Yang *et al.*, 2011). NAC transcription factors also play essential roles in the senescence processes in crop plants. RNA interference studies of wheat *Gpc-B1*, encoding the NAC protein NAM-B1, resulted in delayed leaf senescence and significant reduction in grain zinc, iron, and protein content (Uauy *et al.*, 2006; Waters *et al.*, 2009), and OsNAP, a positive regulator of senescence in rice, affects the grain-filling period and grain yield (Chen *et al.*, 2014; Liang *et al.*, 2014).

The involvement of NAC transcription factors in the regulation of age-dependent/developmental senescence and abiotic stress responses (Nakashima *et al.*, 2012) suggests that these regulators may integrate regulation across different signalling pathways. The NAC-a5 and -a6 subgroups (Shen *et al.*, 2009) contain several NAC transcription factors that have been shown to be involved in the regulation of senescence, for example, AtNAP (Guo and Gan, 2006), TtNAM-B1 (Uauy *et al.*, 2006), ANAC047 (Kim *et al.*, 2014), and OsNAP (Liang *et al.*, 2014), as well as in abiotic stress responses and tolerance (e.g. Xue *et al.*, 2011; Chen *et al.*, 2014).

Barley (*Hordeum vulgare* L.) is an important cereal crop for food, feed, and the beverage industry. To date ~50 NAC genes have been identified in barley which, most likely, constitutes around half of the total number of barley NAC genes (Christiansen *et al.*, 2011). The NAC transcription factor family can be divided into subgroups according to the phylogeny of the N-terminal NAC domain (Shen *et al.*, 2009) with several of these subgroups having conserved functions across monocots and dicots (Christiansen *et al.*, 2011). Nevertheless, the degree of diversification among the NAC genes is high, and there can be distinct differences between closely related homologues in different species (Distelfeld *et al.*, 2012). This emphasizes the importance of full experimental characterization of individual genes.

In this paper we present a detailed characterization of *HvNAC005* (GenBank: AK251058.1; EnsemblPlants: MLOC_65101.1) encoding a barley NAC transcription factor from subgroup NAC-a6 (Christiansen *et al.*, 2011; Kjaersgaard *et al.*, 2011; Christiansenand Gregersen, 2014). Initially, *HvNAC005* was selected for detailed studies because it appeared as the barley NAC gene most closely related to *AtNAP* (Guo and Gan, 2006). Our structural and functional characterization supports HvNAC005 as a strong positive regulator of senescence in barley working in the cross field of hormone and signalling pathways.

## Materials and methods

### Plant growth conditions

Barley plants (*Hordeum vulgare* L.) were grown in a greenhouse with artificial illumination for supplementation when daylight was below 10 klx in order to ensure a day/night cycle of 16/8h. Temperature was only controlled by passive ventilation. No extreme heat periods occurred during the experiments. A standard nutrient solution was supplied via the irrigation system (EC ≤1.8). Most plants were grown in pots of soil (Unimold Substrate, Unimold, Denmark) with the exception of plants for the collection of root samples which were grown on perlite (Nordisk Perlite, Denmark).

### Plant treatments

For abscisic acid (ABA) induction experiments, the second leaf from 3-week-old plants was sprayed until run-off on both sides with a solution of distilled water, 0.05% Triton-X100, and the indicated amounts of 2-*cis*,4-*trans*-abscisic acid (Sigma-Aldrich cat. 862169) dissolved in DMSO. Leaves were sprayed at 08.00 h and harvested after 8h. Dark-induced senescence was achieved by covering the second leaf of 3-week-old plants with tin foil for 3 d.

### Gene expression analysis

Gene expression analysis and screening of transgenic lines were both carried out by quantitative real-time PCR (qRT-PCR). Total RNA was extracted from approximately 100mg of frozen material using the Spectrum™ Plant Total RNA kit (Sigma) according to the manufacturer’s instructions. Synthesis of cDNA and analysis by qRT-PCR were carried out as previously described (Christiansen *et al.*, 2011). The *18S rRNA* and/or *SP2* genes were used for normalization as both were shown to have stable expression across our studies (data not shown). All primers used are listed in Supplementary Table S3 at *JXB* online.

### Generation of transgenic barley lines

The full-length 1068bp *HvNAC005* coding sequence was PCR-amplified from barley ‘Golden Promise’ cDNA and cloned into the pUCE_UBI:USER:NOS_ vector (Hebelstrup *et al.*, 2010) that was used for *Agrobacterium*-mediated transformation of barley ‘Golden Promise’ (Holme *et al.*, 2012). Primers used during cloning are listed in Supplementary Table S1. Regenerated T_0_ lines were screened by qRT-PCR for presence of the transgene, using primer pairs spanning the promoter/coding sequence junction and the coding sequence/NOS terminator junction. Primers are listed in SupplementaryTable S1. Regenerated plants that were negative in tests for the transgene were designated null-transgenic and some of these were used as controls in the microarray and qRT-PCR experiments.

### Phenotyping of transgenic barley lines

Before weighing the total root mass, roots of 3-week-old plants were washed twice in water and blotted dry with paper towels. Root measurements were made on four biological replicates. Dry weight was determined after drying the roots at 42 °C for 24h. Phenology records were based on the appearance of awns from the flag leaf sheath, since most of the spikes did not fully emerge from the sheaths. SPAD measurements of relative chlorophyll concentration were made using a Chlorophyll Meter SPAD-502Plus (Konika Minolta). Measurements were made on 3–5 plants per genotype, at the tip, mid-point, and base of flag leaves, and values were averaged for each leaf. For the time-course of chlorophyll content, measurements were made twice a week (3–5 d intervals) on the two earliest developed tillers of each plant and were started when awns were visible at the top of the leaf sheaths. Since seed quality differed among the lines and affected germination and early development, comparisons were mainly made between over-expression plants and segregating null-transgenic control plants.

### MEME motif identification

Protein sequence motifs were identified using the MEME (Multiple EM for Motif Elicitation) program (Bailey *et al.*, 2009; http://meme-suite.org/tools/meme, last accessed 12 July 2016). The analysis parameters were: maximum width to increase specificity, 10; at least eight sites per motifs; and a cut-off E-value of 1E-25.

### Promoter isolation

The *HvNAC005* promoter was isolated from barley ‘Golden Promise’ using the GenomeWalker^TM^ kit (Clontech) according to manufacturer’s instructions and modified primers listed in Supplementary Table S1.

### Cloning and recombinant protein production

Cloning of GST-tagged HvNAC005 was described previously by Kjaersgaard *et al.* (2011). *Escherichia coli* strain BL21(DE3) was used for expression of GST–tagged recombinant HvNAC005 protein according to standard procedures (Kjaersgaard *et al.*, 2011). Cells were sonicated using a Soniprep 150 instrument (MSE) at amplitude 8μ for 30s, pausing for 1min, and then repeating the sonication process four times. The lysate was then cleared by centrifugation at 12 000 *g* for 20min at 4 °C. The GST fusion protein was purified by affinity chromatography using a 2ml glutathione–Sepharose 4B resin per 250ml of *E. coli* culture using a standard procedure (GE Healthcare). Highly pure GST-HvNAC005(1–172) was produced, and its concentration was quantified by Western blotting using anti-GST antibody (Invitrogen) and anti-rabbit Horse Radish Peroxidase by comparison to a dilution series of recombinant glutathione-*S*-transferase (Sigma).

### Transactivation analysis in yeast

Full-length HvNAC005 and a series of C-terminal deletion derivatives, with or without the NAC domain, were cloned into the DBD vector pGBKT7 (Clontech) using primers listed in Supplementary Table S1. The constructs were transformed into the yeast strain pJ694A and plated onto SD plates without tryptophan (–Trp), plates without tryptophan and histidine (–Trp/–His), and plates without tryptophan, histidine or adenine (–Trp/–His/–Ade). Plates were placed at 30 °C for 5 d before inspection. Subsequently, the cells were grown to an OD_600_ of 0.6–0.8 and diluted to an OD_600_ of 0.4 before 5 µl was spotted on to SD –Trp and SD –Trp/–His plates and incubated at 30 °C for another 5 d.

### Microarray analysis of transgenic plants

Microarray analysis was carried out on 16 samples from 3-week-old barley plants; eight from leaves and eight from roots, comprising three biological replicates from HvNAC005-OE-26 plants, three from null-transgenic lines, and two from wild-type ‘Golden Promise’. Extraction of RNA was as for the gene expression analysis. RNA quality was determined by confirming an OD_260/230_ ratio of >1.8 (NanoDrop ND-1000, Thermo Fisher Scientific) and BioAnalyzer 2100 analysis (Agilent Technologies). All samples had a RIN number ≥6. Two chips of a barley 8×60 Agilent microarray (Kohl *et al.*, 2015) was used for the experiment. Preparation and hybridizations of the samples to the chips (Agilent Technologies, Inc., Santa Clara, USA) were conducted by Source BioScience (Berlin, Germany). Analysis of the microarray data was carried out using the R-based Limma package version 3.16.0 (Smyth, 2005) with the R software version 3.0.1 (R Core Team, 2015), as described previously (Christiansen *et al.*, 2014; Hollmann *et al.*, 2014). Functional analysis of the microarray results made use of the MapMan tool (Usadel *et al.*, 2009). Enrichment of categories among differentially expressed genes was determined using the hypergeometric test in the stats package of R (R Core Team, 2015).

### Electrophoretic Mobility Shift Assay (EMSA)

Affinity purified GST-HvNAC005 were used in EMSAs as previously described (Olsen *et al.*, 2005*b*; Lindemose *et al.*, 2014) using ^32^P-labelled promoter DNA. 1kb (upstream of ATG) of the promoters MLOC_61774 and MLOC_61801 were produced by PCR using genomic barley ‘Morex’ DNA as template and primers listed in Supplementary Table S1. Sequence integrity was checked by sequencing (MWG Operon). Primer MLOC_61774 Fw and MLOC_61801 Fw were individually labelled with T4 polynucleotide kinase and [γ-^32^P] ATP and used in the PCR. To obtain smaller promoter fragments, plasmid pUC19 DNA containing 1kb promoter of MLOC_61774 was double digested with *Eco*RI and *Hin*dIII and ^32^P-labelled using the Klenow fragment and [α-^32^P] dATP. Radio-labelled DNA was gel-purified on 6% native PAGE gels and ethanol-precipitated before use in EMSA. The DNA concentration was roughly kept at 3nM. The sample volume was 30 μl and affinity purified NAC proteins were tested in the range from 5–200ng per sample. 200ng of GST tag was used as the negative control. The binding reactions were incubated for 30min at room temperature (22 °C) and subsequently separated by native PAGE (6%) using 1× TRIS borate EDTA as running buffer. The gels were exposed to phosphor storage screens and data collected by a Molecular Dynamics STORM 860 PhosphorImager scanner. ImageQuant software version 5.2 (Molecular Dynamics) was used to visualize the EMSAs.

## Results

### Transcriptional regulation of HvNAC005

**Fig. 1.**
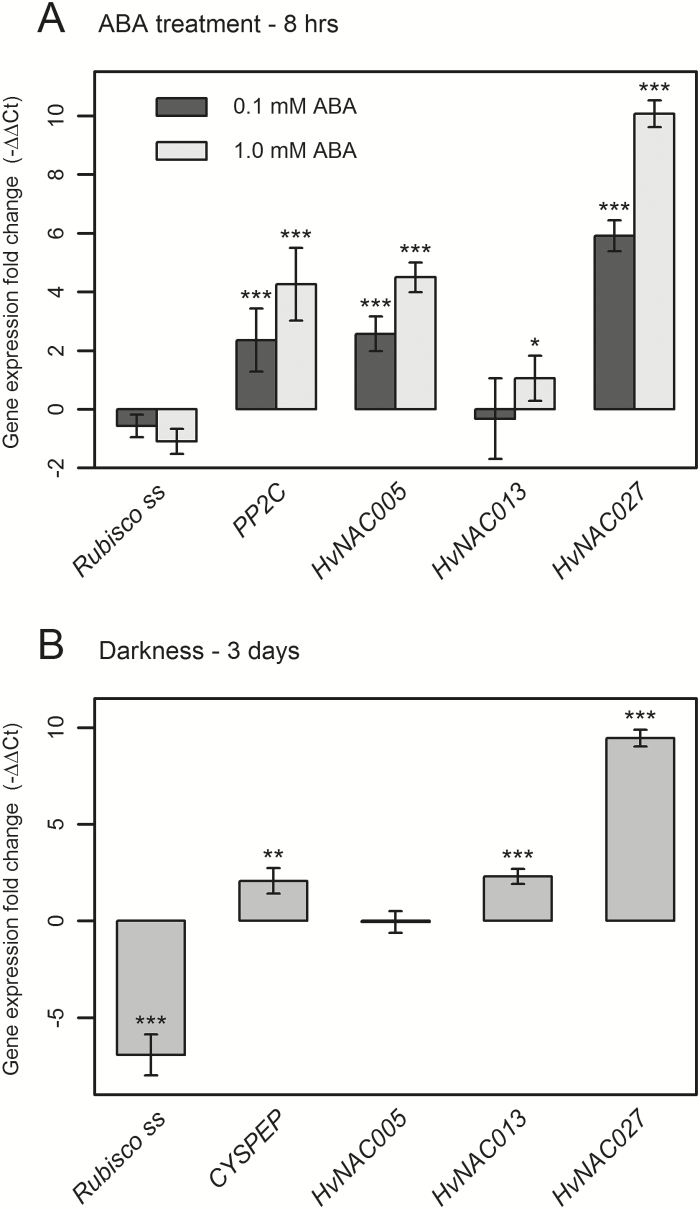
*HvNAC005* expression levels, as determined by qRT-PCR, following ABA treatment and darkness in barley ‘Golden Promise’ plants. The qRT-PCR results were normalized to the expression level of *18S rRNA*. Gene expression levels (–∆∆Ct, i.e. log_2_ scale) was determined relative to untreated control leaves. (A) ABA treatment of 2nd leaves of 3-week-old plants with either 0.1 or 1.0mM ABA. (B) Three days of dark treatment, i.e. coverage with tin foil, of 2nd leaves of 3-week-old plants. – *Rubisco ss, Rubisco small subunit* (GenBank: AB020943); *PP2C, protein phosphatase 2C* (GenBank: BM816007); *HvNAC005* (GenBank: AK251058); *HvNAC013* (GenBank: AK376297); *HvNAC027* (GenBank: FR819765); *CYSPEP*, *papain cysteine peptidase* (GenBank: AK358908.1). Error bars represent SE. */**/***, *P* <0.05/0.025/0.001 (*t* test, R software version 3.0.1, R Core Team, 2015).

Previous gene expression studies showed that barley members of the NAC-a5 and -a6 phylogenetic subgroups, *HvNAC005*, *HvNAC023, -27, -29*, and -*30*, form a group of ABA responsive genes with relatively early up-regulation during senescence that tends to level off at later stages (Christiansen *et al.*, 2011; Christiansen and Gregersen, 2014). In the present study, *HvNAC005* had a moderate response to ABA compared with the strong response of *HvNAC0027*, and the low response of *HvNAC013* (Fig. 1A). An ABA-inducible protein phosphatase gene (GenBank: BM816007) was included as a positive control. In contrast, *HvNAC005* showed no transcriptional up-regulation during dark-induced senescence (Fig. 1B), although other barley NAC genes and a papain-like cysteine peptidase SAG (*CYSPEP;* GenBank: AK358908.1) were up-regulated by the treatment. This is in contrast to the *ONAC58/OsNAP* orthologue of *HvNAC005*, which was previously shown to be dark-induced (Liang *et al.*, 2014).

### The NAC-a5 and -a6 subgroups (‘NAP’)

**Fig. 2.**
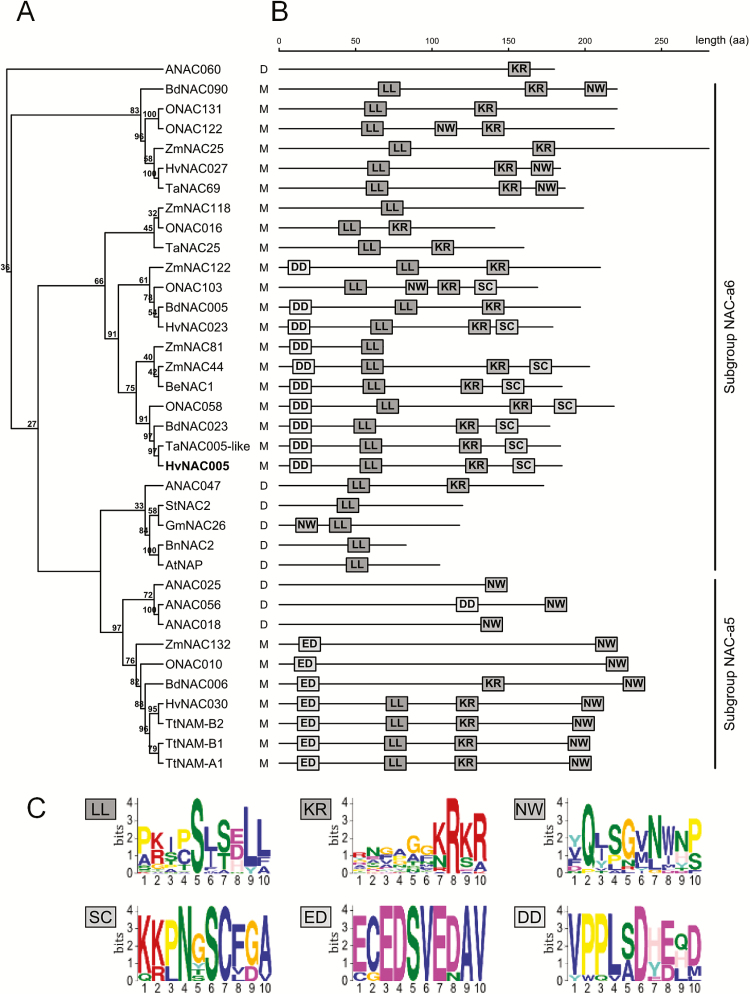
Features of the NAC domain proteins in the subgroups NAC-a5 and -a6 (Shen *et al.*, 2009). (A) Phylogenetic tree of HvNAC005-related proteins belonging to the NAC-a5 and -a6 subgroups (Supplementary Table S2). The tree was based on the N-terminal NAC domains of the proteins and constructed using the ClustalX software. ANAC060 (subgroup NAC-d) was included as an outgroup. D, dicot; M, monocot. (B) C-terminal parts corresponding to the NAC domains in (A), highlighting conserved MEME amino acid motifs. (C) WEBlogos of the six MEME motifs shown in (B). The height of a letter in the WebLogo reflects its relative frequency at the given position in the motif. (This figure is available in colour at *JXB* online).

A phylogenetic analysis clearly places HvNAC005 in subgroup NAC-a6 of the NAC domain protein family, using the phylogenetic grouping by Shen *et al.* (2009) (Fig. 2A). Subgroup a6, together with subgroup a5, was denoted the ‘NAP’ group in earlier phylogenetic analyses (Ooka *et al.*, 2003). The basis for subdividing the ‘NAP’ group is clear from the tree of 35 proteins closely related to HvNAC005 in Fig. 2A, which was substantiated by analysis of conserved motifs in the C-termini. The C-termini are very variable across the NAC domain family; however, they are characterized by short group-specific sequence motifs (Jensen *et al.*, 2010), also evident when analysing HvNAC005 and its homologues from the NAC-a5/-a6 subgroups. MEME analysis of the C-termini resulted in the identification of six well-defined short amino acid motifs (Fig. 2C). The pattern of motifs across the two subgroups aligned well with the phylogenetic relationships in Fig. 2A, in particular, with respect to dicot and monocot subclades (Fig. 2B).

**Fig. 3.**
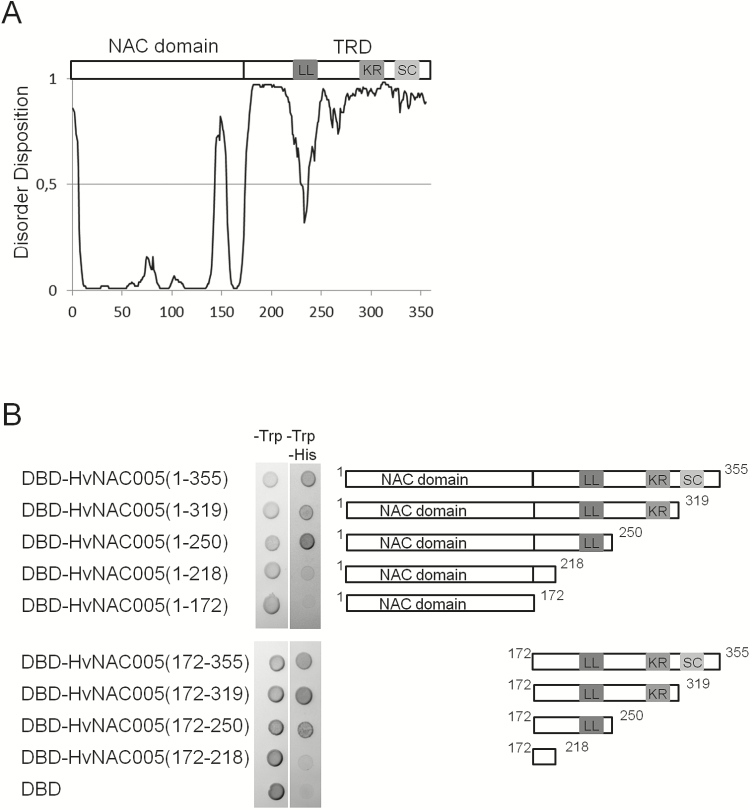
Structure and molecular function of HvNAC005. (A) Predicted structure of HvNAC005. Top, Schematic structure of HvNAC005 drawn to scale with the sequence motifs LL, KR, and SC (Fig. 2C) shown in the C-terminal region. Bottom, PONDR-FIT (Xue *et al.*, 2010) protein intrinsic disorder prediction of HvNAC005 with a threshold for disorder assigned to values ≥0.5. (B) Yeast transactivation assay. Fusion proteins of GAL4 DNA-binding domain (DBD) and HvNAC005 fragments (right) were expressed in yeast and screened for transactivation activity using the *HIS3* reporter gene. Yeast cells were streaked onto plates lacking tryptophan (–Trp) to check for transformation and on plates lacking both tryptophan and histidine (–Trp–His), which allowed analysis for transactivation activity. Empty pGBKT7 encoding the GAL4 DBD served as a negative control.

The LL and KR motifs were found across both NAC-a subgroups and the KR motif even in the ANAC060 outgroup sequence. The subgroup a5 was characterized by the distal NW motif. It is noteworthy that AtNAP, involved in senescence regulation in Arabidopsis (Guo and Gan, 2006), appeared truncated compared with most of the other subgroup a5/a6 proteins and only showed the highly conserved LL-motif in its C terminal sequence. The monocot subclades neighbouring HvNAC005 were characterized by two distal conserved motifs, KR and SC, and the proximal DD motif. Similarly, the monocot sequences of subgroup a5 were characterized by the distal NW and the proximal ED motifs. Both of these proximal motifs are rich in D/E amino acids, typical of disordered regions of proteins (Chou and Wang, 2015). By contrast, the LL motif mapped to a region of predicted order in the overall disordered HvNAC005 C-terminus (Kjaersgaard *et al.*, 2011; Fig. 3A) and it may represent a preformed structural element participating in protein–protein interactions (Kragelund *et al.*, 2012).

### Transcriptional activity of HvNAC005

HvNAC005 has a structure typical of NAC domain proteins and is predicted to be a transcription factor with the intrinsically disordered C-terminal domain functioning as a transcription regulatory domain (Jensen *et al.*, 2010). In previous work, HvNAC005 did not show abilities to activate transcription when tested in yeast under standard conditions, leading to the suggestion that HvNAC005 may, instead, function as a transcriptional repressor (Kjaersgaard *et al.*, 2011). However, since closely related NAC proteins from both Arabidopsis (Guo and Gan, 2006) and bamboo (Chen *et al.*, 2011) were found to activate transcription, additional yeast one-hybrid experiments were performed with HvNAC005. In the study by Kjaersgaard *et al.* (2011), the ability to activate transcription was measured in the absence of both histidine and adenine in the selective growth plates. When adenine was added to the plates, both full-length HvNAC005, HvNAC005(1–355), and the HvNAC005 C-terminus, HvNAC005(172–355), were able to activate transcription of the reporter gene *HIS3,* as measured by the ability of transformed yeast to grow in the absence of histidine (Fig. 3B). By contrast and, as expected, the NAC domain, HvNAC005(1–172) alone was unable to activate transcription.

In order to analyse whether the regions containing conserved C-terminal motifs are of importance for the ability of HvNAC005 to activate transcription, C-terminal truncations with stepwise motif removal were analysed in yeast. Removal of the SC- and the KR-motifs from both full-length and C-terminal HvNAC005 did not affect the ability to activate transcription (Fig. 3B). By contrast, removal of the LL-motif abolished activity in both cases. This demonstrated the dependence of transcriptional activity on a region with a short amino acid motif that was predicted to have structure despite being embedded in a disordered protein region (Fig. 3A).

### Promoter region of HvNAC005

The genome walking technique was used to clone and sequence an upstream genomic sequence (1787bp, EMBL: LN886704) of *HvNAC005*, which was 99% identical to the upstream genomic sequence of the locus MLOC_65101.1 (EnsemblPlants) in barley ‘Bowman’ (http://plants.ensembl.org/Hordeum_vulgare/, last accessed 12 July 2016). The 600 bases upstream of ATG were aligned for the five closest members of the NAC-a6 subgroup from barley, wheat, *Brachypodium*, maize, and rice (see Supplementary Fig. S1 at *JXB* online). The TATA box of the promoters were found around position –200 and the transcription start is predicted to be at position –173 in *BdNAC023* (Bradi1g63600.1). The alignment showed high conservation among the five species but also diversifications reflecting general phylogenetic relationships. An ABRE related sequence motif, CACGTG (G-box), generally found in ABA regulated genes (Zhang *et al.*, 2005), was present around position –415 (barley) for all five sequences, in addition to another ACGT-containing motif 40 bases downstream of the former motif (except in maize that had an additional ACGT motif starting at position –356). A conserved region starting around 14 bases down-stream of the G-box, and including the latter ACGT motif, might be an ABRE coupling element often found downstream of ABRE motifs (Shen *et al.*, 2004). In barley, wheat, and *Brachypodium*, a third ACGT containing motif was present around position –340 (barley). Two ACGT motifs were found in promoters for most of the monocot species across the NAC-a5/-a6 transcription factor genes shown in Fig. 2; however, not for *AtNAP*, which only has one ACGT containing motif in its promoter sequence, at position –393 relative to ATG (data not shown). This difference might reflect differences in the ABA inducibility of these genes, for which many of the monocot genes (e.g. Xue *et al.*, 2006; Christiansen *et al.*, 2011; and this study) have shown strong responses, whereas *AtNAP* is only moderately induced by ABA (Guo and Gan, 2006). Interestingly, ACGT is also present in the core of the accepted NAC-binding site model T[G/A]CGT (Olsen *et al.*, 2005*a*; Lindemose *et al.*, 2014).

### Phenotyping of HvNAC005 over-expressing plants

**Fig. 4.**
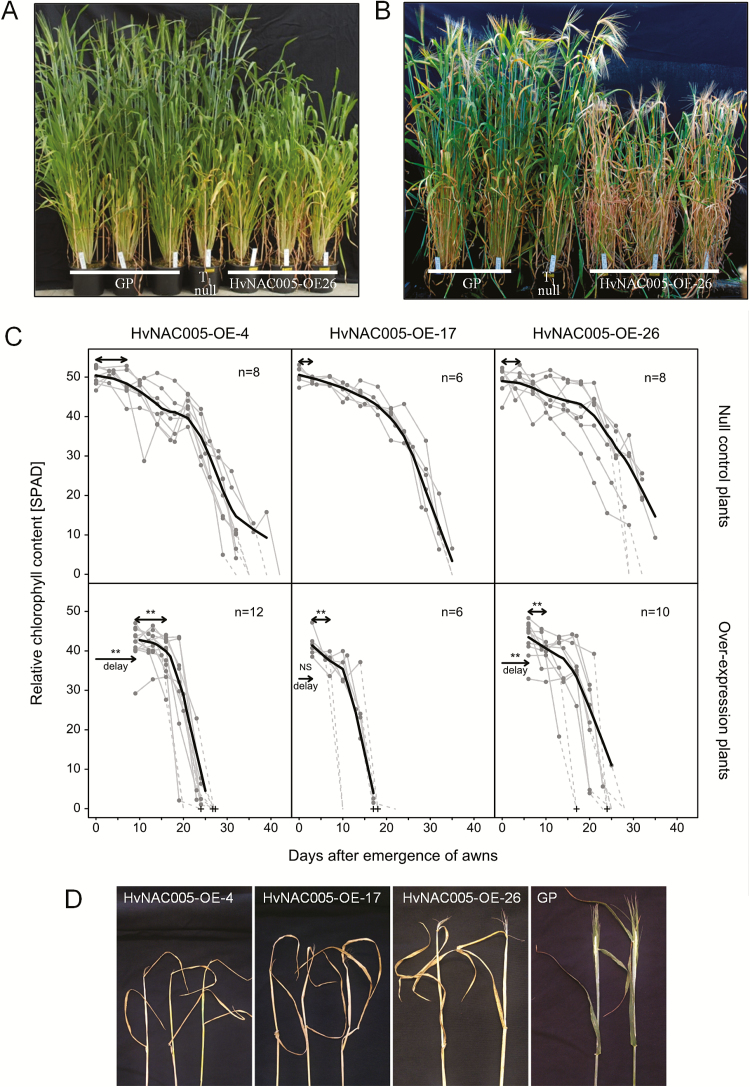
Characterization of T_1_ *HvNAC005* over-expression plants. (A) Phenotype of plants at the heading stage, 3 months after sowing, line HvNAC005-OE-26. (B) Senescence phenotype of plants 4 months after sowing, line HvNAC005-OE-26 compared with control plants. (C) Relative chlorophyll content (SPAD values=mean of three measurements per leaf) in flag leaves of three independent transgenic lines HvNAC005-OE-4, -17, and -26, from greenhouse grown plants. Measurements were made twice a week on the two earliest developed tillers of each plant and started when awns were visible at the top of the leaf sheaths. The upper panels show results for null-transgenic control plants and the lower panels for the *HvNAC005* over-expression plants. Values for individual leaves are shown in grey. The bold line is a lowess smooth regression line through the data points (R software version 3.0.1, R Core Team 2015), excluding the data points from dead, shrivelled leaves. SPAD values from these leaves could not be obtained, and they were set to SPAD=0. These zero data points for dead leaves are connected to the last data point from the living leaf by a dashed line. The starting time was scaled to start at time=0 for all of the control plants, causing the data points for different leaves not to be completely equidistant. For the over-expression plants, the starting time was shifted by the average delay in awn emergence compared with the respective control plants, indicated by a uni-directional arrow. Asterisks indicate whether this delay was statistical significant, *P* ≤0.05 (*t* test, R software version 3.0.1). Bi-directional arrows indicate the first two sampling time points used to compare the initial chlorophyll content between control and over-expression plants. Asterisks indicate a statistical significant difference in this comparison, *P* ≤0.05 (*t* test, R software version 3.0.1, R Core Team, 2015). (D) Senesced/dead tillers of the three lines HvNAC005-OE-4, -17, and -26 sampled 94 d after sowing, compared with tillers of ‘Golden Promise’ of a similar age. n, Number of flag leaves (number of plants is n/2). **+**, Indicates tillers shown in Fig. 4D; NS, not statistical significant. GP, ‘Golden Promise’ control plants; T_1_ null, null-transgenic T_1_ plants. (This figure is available in colour at *JXB* online).

To investigate the function of HvNAC005 in barley, we generated transgenic lines constitutively over-expressing the full-length coding sequence. Twelve positive transgenic lines were generated, all exhibiting elevated levels of *HvNAC005* transcripts (Supplementary Fig. S2). Plants from these lines were all stunted in comparison to the wild-type plants and null-transgenic lines and they showed precocious senescence (shown for line HvNAC005-OE-26 in Fig. 4A, B). The severe phenotype caused poor seed setting, with smaller spikelets and fewer filled seeds. Only three lines with sufficient seed setting could be used for further analysis: HvNAC005-OE-4, -17, and -26. We have not been able to identify homozygous lines, suggesting that high levels of *HvNAC005* expression are detrimental to plant development.

T_1_ plants from the three lines were characterized in a time-course experiment (Fig. 4C). Senescence of the flag leaves were monitored by measuring relative chlorophyll content (SPAD). To reveal any delays in development, tillers with visible awns were counted throughout the experiment. This was used instead of spike emergence, since most of the spikes in transgenic plants remained within the leaf sheath. For all three lines, there was a delay in the appearance of awns in the first two developed tillers, when compared with the null-transgenic control plants, although this delay was only statistical significant for lines -4 and -26. The same pattern of delay in development of the transgenic lines was reflected in the number of days after sowing until half of the final number of tillers with visible awns had appeared (Supplementary Table S4). Overall, OE plants showed a delay compared with ‘Golden Promise’ control plants, whereas null-transgenic plants were similar to ‘Golden Promise’. The final total numbers of tillers were in the same, quite variable, range for all plants, showing only a slight significant reduction for HvNAC005-OE-26 plants (Supplementary Table S5). The initial chlorophyll content, from the first two measurements after awn appearance, was significantly lower in HvNAC005-OE plants compared with the respective control plants (Fig. 4C, bi-directional arrows). Flag leaves of the first two developed tillers in HvNAC005-OE plants had a steep decrease in chlorophyll content compared with control plants. These tillers, including the premature spikes, had all died within 20 d after the appearance of awns, whereas it took close to 30 d before similar tillers in the control plants started to die, reflecting precocious senescence in the HvNAC005-OE plants (Fig. 4D). Seed setting of HvNAC005-OE plants was very poor for all three lines in this experiment. Only a few late-developed spikes produced a limited number of seeds, whereas most of the spikes either had already died within the leaf sheath or produced infertile spikelets.

The line HvNAC005-OE-26 was studied for its root phenotype. Root mass was visibly reduced in 4-week-old transgenic plants, with both root fresh weight (Supplementary Fig. 3S) and dry weight (data not shown) of the total root system around half of the wild-type weight.

### Barley Agilent microarray analysis of HvNAC005 over-expressing plants

To investigate the regulatory networks of HvNAC005, an Agilent microarray study was performed on root and leaf tissues from three 3-week-old *HvNAC005* over-expressing plants (line HvNAC005-OE-26), and from three null-transgenic plants and two wild-type ‘Golden Promise’ plants as control lines. Raw data from the experiment are available from ArrayExpress (http://www.ebi.ac.uk/arrayexpress/, last accessed 12 July 2016, accession # E-MTAB-3920). In general, the differential expression between transgenic and non-transgenic plants was more pronounced in roots than in leaves and, in general, the fold changes observed were relatively small. Hence, only 716 probes showed fold changes higher than 2 among the top 2841 selected probes (see below), and only 110 showed fold changes higher than 4. However, in the contrast between HvNAC005-OE-26 roots and the average of wild-type ‘Golden Promise’ and the null-transgenic line, a cut-off *P*-value <0.05 selected 2841 probes as differentially expressed, supported by a correlation between differential expression in transgenic roots and transgenic leaves compared with the combined control. The redundancy of probe sequences was reduced using the procedure described by Christiansen and Gregersen (2014), which resulted in a list of 2630 differentially expressed genes out of 26 826 genes in total. There were slightly more up-regulated (1442), compared with down-regulated (1235) genes among the differentially expressed genes.

**Fig. 5.**
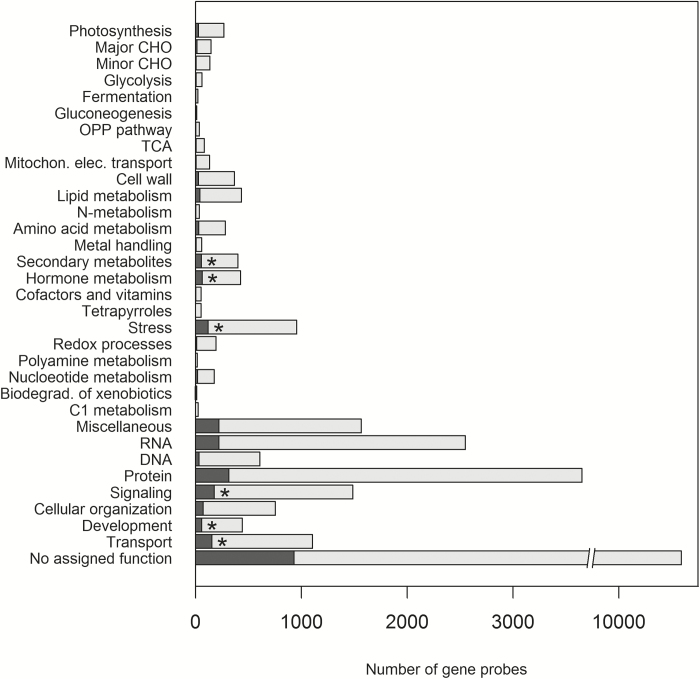
Representation of genes in the MapMan top BINs for all genes (dark+light grey) and the 2630 differentially expressed genes (dark grey), respectively, in the Agilent microarray experiment for *HvNAC005* over-expressing plants, line HvNAC005-OE-26. Asterisks indicate significant overrepresentation among differentially expressed genes for individual categories determined using a hypergeometric test (R software version 3.0.1, R Core Team, 2015).

**Fig. 6.**
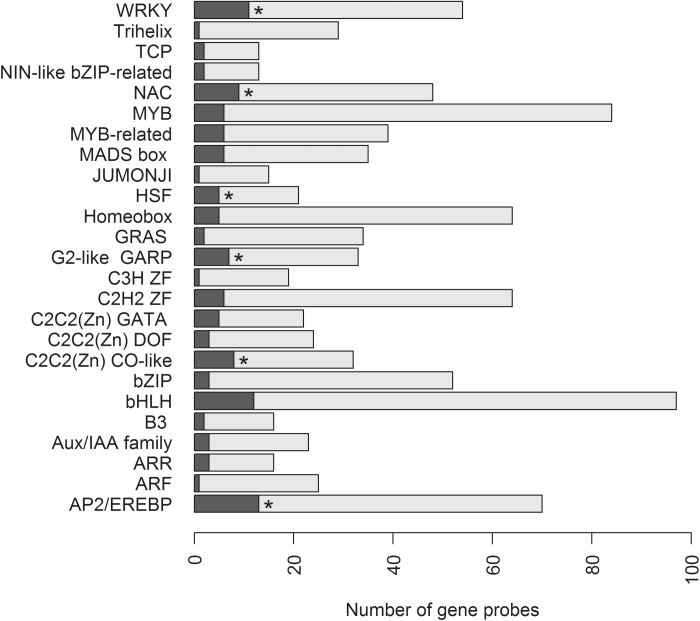
Representation of genes in the MapMan transcription factor BINs for all genes (dark+light grey) and the 2630 differentially expressed genes (dark grey), respectively, in the Agilent microarray experiment for *HvNAC005* over-expressing plants, line HvNAC005-OE-26. Asterisks indicate significant overrepresentation among the differentially expressed genes for individual categories using a hypergeometric test (R software version 3.0.1, R Core Team, 2015).

MapMan analysis (Supplementary Table S6) of the 2630 genes showed a significant overrepresentation of genes in the BINs for secondary metabolites (BIN 16), hormone metabolism (BIN 17), stress (BIN 20), signalling (BIN 30), development (BIN 33), and transport (BIN 34) (Fig. 5), determined by a hypergeometric test (R Core Team, 2015). Regarding the signalling BIN (30), there is an overlap with the protein BIN (29), with respect to signalling via the action of protein kinases. In relation to our qRT-PCR results below, there is, however, no overlap in the established BINs regarding protein phosphatases which are also important in the signalling processes and of particular interest in the context of HvNAC005 with its high homology to AtNAP that has been shown to interact with SAG113, a protein phosphatase 2C (PP2C) (Zhang and Gan, 2012). Hence, the protein BIN were searched for genes having descriptions that included ‘PP2C’ or ‘phosphatase 2C’ (Supplementary Table S6). These genes (in total 17) did indeed show an overrepresentation among differentially expressed genes, even though the protein top BIN did not. Furthermore, the top four up-regulated *PP2C* genes encoded proteins closely related to SAG113. Another BIN of interest is that for transcription factor genes, included in the RNA BIN (27). Overall this BIN did not show overrepresentation among differentially expressed genes; however, when analysing transcription factor sub-BINs, we observed overrepresentation of genes from the WRKY, NAC, HSP, C2C2-(Zn) CO-like, and AP2/EREBP families (Fig. 6).

The BINs showing overrepresentation among differentially expressed genes included both up- and down-regulated genes, but two groups, stress (BIN 20) and signalling (BIN 30), showed a significant deviation from this overall distribution in having a high representation of down-regulated genes. Analysis of sub-BINs showed that genes involved in biotic stress predominated among down-regulated genes in the stress BIN and that genes related to receptor kinases predominated among down-regulated genes in the signalling BIN (Supplementary Table S6).

### Validation of microarray data using qRT-PCR

A number of genes were selected for qRT-PCR tests for differential expression, based on the microarray results and also on knowledge about SAGs in barley (Christiansen and Gregersen, 2014) and in other species (Supplementary Table S3). We performed experiments both with leaf tissue from 2-week-old seedlings (lines HvNAC005-OE-17 and -26) and root tissue from single seeds germinated on moist filter paper (lines HvNAC005-OE-17 and -4). In both cases it was difficult to obtain transgenic material since low seed setting in *HvNAC005* over-expressing plants meant that very few of the viable seeds were, in fact, transgenic.

**Fig. 7.**
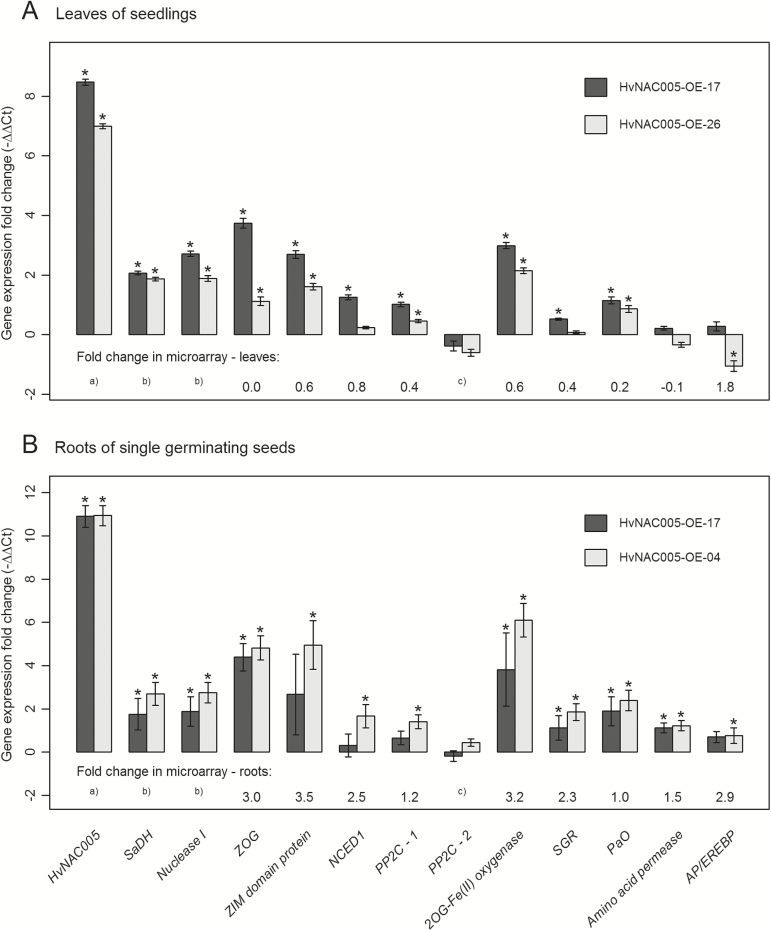
Validation by qRT-PCR of gene expression levels in barley *HvNAC005* over-expressing lines for selected SAGs and genes selected from the microarray experiment (Supplementary Table S3). Ct values were normalized to *18S rRNA* expression and the results (–∆∆Ct, i.e. log_2_ scale) are shown relative to expression levels in null-transgenic lines as controls. Where available, the fold change in the microarray experiment is shown in the bottom of the figures. (A) Results from leaves of 2-week-old seedlings. (B) Results from 3-d-old roots of single seeds germinated on moist filter paper. *SaDH, saccharopine dehydrogenase* (GenBank: AJ508229.2); *Nuclease I, BnucI* (GenBank: AB028448.1); *ZOG,* putative *zeatin O-glucosyltransferase* (GenBank: AK364192.1); *ZIM domain protein, jasmonate ZIM domain-containing protein* (GenBank: AK358513.1); *NCED1: 9-cis-epoxycarotenoid dioxygenase* (GenBank: AK365103.1); *PP2C–1, protein phosphatase 2C* (GenBank: AK367469.1); *PP2C–2, protein phosphatase 2C* (GenBank: AK374059.1); *2OG-Fe(II) oxygenase, oxoglutarate/iron-dependent dioxygenase* (GenBank: AK355337.1); *SGR, staygreen protein* (GenBank: AK361634.1); *PaO, pheophorbide* a *oxygenase* (GenBank: AK358479.1); *Amino acid permease, amino acid permease* (GenBank: AK361903.1); *AP/EREBP, APETALA2 and ethylene responsive element binding protein* (GenBank: AK374826.1).^( a)^ The microarray probe for *HvNAC005* was in the 3′-UTR and, hence, did not detect the transcript of the *HvNAC005* transgene.^( b)^ The *SaDH* and *Nuclease I* genes were not represented among the microarray probes.^(c)^ The signal for the *PP2C-2* microarray probe was filtered out during data analysis. Error bars represent SE. *, *P* <0.05 (Limma test; Smyth, 2005).

In both experiments (Fig. 7), two SAGs encoding a nuclease I and a saccharopine dehydrogenase consistently showed strong up-regulation, as clear markers of senescence. Unfortunately, there were no probes representing these genes in the Agilent chip.

Since up-regulated genes in hormone metabolism and signalling categories were of interest from the microarray results, genes from these categories were among those selected for analysis by qRT-PCR: *ZOG* (*zeatin O-glucosyltransferase*) putatively involved in cytokinin metabolism; a *ZIM domain protein* (*jasmonate ZIM domain protein*), putatively involved in jasmonate signalling; *NCED1* (*9*-cis*-epoxycarotenoid dioxygenase 1*), involved in ABA biosynthesis; and a *PP2C* (*protein phosphatase2C*), putatively involved in ABA signalling. Up-regulation of all these genes was confirmed in the qRT-PCR experiments supporting the hypothesis that HvNAC005 works in a cross field of signalling pathways involving different hormones. Furthermore, since AtNAP has been shown to interact directly with a PP2C protein (Zhang and Gan, 2012), we tested two *PP2C* genes, one that was among the up-regulated genes (*PP2C-1*) and another (*PP2C-2*) that was not. This difference in expression was confirmed in the qRT-PCR experiments (Fig. 7). The *2OG-Fe(II) oxygenase gene* was included because it was one of the most strongly up-regulated genes in the microarray but, unfortunately, no specific function can be ascribed to this gene. The genes *SGR* (*staygreen protein;* Park *et al.*, 2007) and *PaO* (*pheophorbide a oxygenase;* Pružinská *et al.*, 2003) are both involved in chlorophyll breakdown and degradation. Confirmation of their up-regulation in *HvNAC005* over-expressing plants supports a role of HvNAC005 in this catabolic process which is an intrinsic part of the general senescence process.

HvNAC005-OE-26 showed lower expression level of the *HvNAC005* transgene than other lines (Supplementary Fig. S2), and this was reflected in the expression levels of other up-regulated genes when comparing expression levels in the leaves of this line and line HvNAC005-OE-17 (Fig. 7A). In conclusion, qRT-PCR showed variations in the validation of the microarray results across different tissues/experiments but, in general, it substantiated the differential expression of categories of genes as described for the microarray experiment. In addition, it showed the up-regulation of two strong senescence marker genes encoding a nuclease I and a saccharopine dehydrogenase.

### Analysis of HvNAC005 DNA binding in Electrophoretic Mobility Shift Assays

The number of verified NAC binding targets is still limited. However, in previous studies, several NAC proteins from Arabidopsis have been shown to bind to DNA containing the NAC binding site (NACBS) core motif TTNCGT and variations thereof (Olsen *et al.*, 2005*a*; Jensen *et al.*, 2010; Welner *et al.*, 2012; Lindemose *et al.*, 2014). Indeed, HvNAC005 was previously shown to bind to this core motif (Kjaersgaard *et al.*, 2011).

For EMSA analysis of barley promoter sequences corresponding to up-regulated genes in our microarray experiment and the qRT-PCR validation, we selected promoters of the *ZIM domain protein* (EnsemblPlants: MLOC_61774.1) and the *ZOG* gene (EnsemblPlants: MLOC_61801.1). The promoters of both genes do indeed contain several potential NACBSs with the core motif present in both DNA strand orientations (Supplementary Fig. S4). It is noteworthy that palindromic binding sites containing both CGT and ACG are better binding sites than a single CGT or ACG (Olsen *et al.*, 2005*a*).

**Fig. 8.**
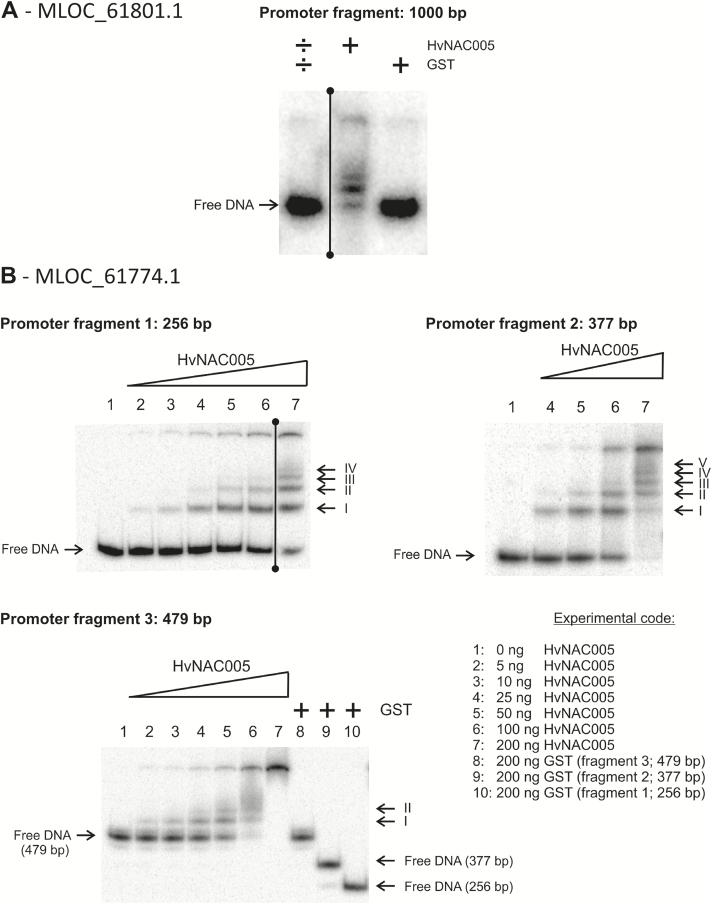
EMSA assays with the DNA binding NAC domain of HvNAC005 and the promoter sequences of two genes selected from the genes up-regulated in the microarray experiment. Glutathione-*S*-transferase (GST) was included as a negative control. (A) EMSA results with the 1000bp promoter of the ZOG gene (EnsemblPlants: MLOC_61801.1; GenBank: AK364192.1). A number of other NAC transcription factors were tested on this promoter fragment but were, for the sake of clarity, excluded from the figure. (B) EMSA results with three promoter fragments of a ZIM domain protein (EnsemblPlants: MLOC_61774.1; GenBank: AK358513.1). EMSA assays were performed at least in triplicate. Between 100 and 200ng HvNAC005 we observed 'smear'and precipitation in the wells, which obscured the number of possible protein–DNA complexes. Thus, to obtain the best qualitative result, the best two titrations were merged.

After performing initial tests on 1kb PCR fragments of both promoters (shown for MLOC_61801 in Fig. 8A), it was evident that HvNAC005 binding sites exist in the two 1kb promoters (Supplementary Fig. S4). To acquire a better resolution, the cloned 1kb promoter of MLOC_61774 was digested with restriction enzymes giving rise to three DNA fragments. Using binding conditions described previously (Olsen *et al.*, 2005*b*), the titration of HvNAC005 elicited multiple well-defined and sharp gel shifts and super-shifts for all three DNA fragments in a concentration-dependent manner (Fig. 8B). The purified GST tag in itself did not produce any gel-shifts in the concentration range analysed, emphasizing that the HvNAC005-DNA interaction is sequence-specific and not due to unspecific low-affinity binding. In addition, the different protein–DNA complexes observed in EMSAs (named I to IV in Fig. 8B) showed relatively large differences in affinities, highlighting the potential importance of sub-optimal binding sites in differential gene expression. Interestingly, inspections of Table 3 (experimentally observed NAC binding sites) and Table 4 (putative NAC binding sites in barley promoters) in Christiansen and Gregersen (2014) and Table 1 (DNA-binding by stress and senescence associated NAC proteins) in Jensen and Skriver (2014) clearly demonstrate this point. Hence, it is evident that a vast variety of different NAC binding sites exists which has impact on protein–DNA affinities and thus expression levels of target genes. In conclusion, the EMSA results demonstrate that the DNA binding of HvNAC005 to the promoters of MLOC_61774.1 and MLOC_61801.1 could potentially regulate the expression of these two barley genes as observed in *HvNAC005* over-expression plants.

## Discussion

Senescence has been extensively studied in the model plant Arabidopsis (Guo *et al.*, 2004; Breeze *et al.*, 2011; Kim *et al.*, 2014), whereas knowledge of the molecular mechanism of senescence in crop plants is more limited. Since functional divergence of orthologous NAC transcription factors may hamper the translation of results from model to crop plants (Distelfeld *et al.*, 2012), specific studies of individual NAC transcription factors are necessary. *HvNAC005* has previously been associated with both senescence and ABA-responses based on its expression patterns (Christiansen *et al.*, 2011; Christiansen amd Gregersen, 2014), and this was confirmed here. Thus, over-expression of *HvNAC005* resulted in a stunted and precocious senescence phenotype.

HvNAC005 belongs to the subgroup NAC-a6 (Shen *et al.*, 2009) containing several members such as TaNAC069, AtNAP, BeNAC, and OsNAP, for which roles in senescence have been demonstrated (Guo and Gan, 2006; Xue *et al.*, 2006; Chen *et al.*, 2011; Liang *et al.*, 2014). As for *TaNAC069, AtNAP,* and *OsNAP*, over-expression of *HvNAC005* resulted in delayed development of the plant but also an early senescence phenotype with early withering of leaves and whole tillers. Xue *et al.* (2011) stated that there was no change in phenotype of *TaNAC69* over-expression in wheat, except for a reduction in grain weight. However, we tested two lines of these plants in our own greenhouse and observed a premature senescence phenotype comparable with that of *HvNAC005* in barley. In rice, Chen *et al.* (2014) reported no change in the phenotypes of *OsNAP* over-expression lines, in contrast to results reported by Liang *et al.* (2014). The discrepancies in the effects on phenotypes might relate to the use of different promoters in the various studies. The strong maize ubiquitin promoter used in our study may have resulted in higher expression levels than with other promoters. The over-expression of *HvNAC005* resulted in reduced root mass and poor seed setting in barley, which appeared much stronger than reported for rice and wheat.

No transcriptional up-regulation of *HvNAC005* was seen during dark-induced senescence (Fig. 1), which was in contrast both to the senescence-associated *HvNAC013* and *HvNAC027* genes and to *OsNAP* in rice (Liang *et al.*, 2014). Dark-induced senescence was previously shown to differ considerably from natural developmental senescence with respect to gene expression profiles (Buchanan-Wollaston *et al.*, 2005), and the regulation of this process might be a point of divergence across species. Subtleties in regulation patterns among the closely related NAC-a genes might be related to diversification of the promoter regions of the genes. The differences among even closely related orthologues are quite extensive, with only some conserved ABRE containing motifs (Supplementary Fig. S1). Functional similarities and differences have previously been reported for AtNAP and OsNAP. Both AtNAP and OsNAP function through ABA-and SAG-dependent pathways to mediate chlorophyll degradation (Yang *et al.*, 2014; Liang *et al.*, 2014). However, OsNAP inhibited and AtNAP promoted ABA-biosynthesis. In addition, OsNAP did not activate transcription from the promoters of *OsPP2C09* and *OsPP2C68*, which are homologues of *SAG113*, an important target gene of AtNAP, encoding a PP2C (Zhang and Gan, 2012). This shows that OsNAP is likely to mediate its ABA-associated function in rice in a manner different from the function of AtNAP in the ABA–AtNAP–SAG113 chain in Arabidopsis (Zhang and Gan, 2012). We did not test the direct interaction of HvNAC005 with PP2C proteins, but the up-regulation of several *PP2C* genes in the *HvNAC005* over-expression plants suggests involvement of these proteins in the regulatory network of HvNAC005.

The function of OsNAP as a positive regulator may involve jasmonic acid (Zhou *et al.*, 2013), but a similar involvement has so far not been shown for other related NAC transcription factors. We found a possible interaction with jasmonate signalling pathways via the up-regulation by HvNAC005 of a ZIM-domain protein encoding gene (Fig. 7). These proteins repress jasmonate induced genes, but are also induced themselves by jasmonate (Wasternack and Hause, 2013). This could indicate an involvement of HvNAC005 in a complex cross-talk between the signalling pathways of different hormones. This hypothesis is further substantiated by the induction of a putative *cytokinin glucosyltransferase, ZOG*, in *HvNAC005* over-expressing plants. Up-regulation of *ZOG* genes have been shown to take place during senescence in wheat (Song *et al.*, 2012).

HvNAC005 has a typical NAC protein structure consisting of a DNA-binding NAC domain and an intrinsically disordered transcription regulatory domain. HvNAC005 is likely to target the consensus NAC binding site [G/A]CGT (Olsen *et al.*, 2005*b*; Lindemose *et al.*, 2013), and we showed that the promoter of MLOC_61744, which encodes the putative ZIM domain protein, contains several putative NAC binding sites and that a recombinant version of HvNAC005 NAC domain binds to promoter fragments containing these sites. Therefore, MLOC_61744 is likely to be a direct target gene of HvNAC005.

MapMan analysis of the 2630 genes identified as differentially expressed in our microarray analysis of HvNAC005-OE showed significant overrepresentation of genes in BINs for secondary metabolites, hormone metabolism, stress, signalling, development, and transport, in accordance with an association with senescence (Breeze *et al.*, 2011). Interestingly, although the RNA BIN overall did not show a high proportion of differentially expressed genes, numerous transcription factor genes from the WRKY, NAC, HSP, C2C2-(Zn) CO-like, and AP2/EREBP families were observed to have altered expression when analysing transcription factor sub-BINs, in alignment with studies in Arabidopsis (Breeze *et al.*, 2011).

HvNAC005 was shown to function as a transcriptional activator in yeast, like other NAC transcription factors of the NAC-a6 subgroup (Jensen *et al.*, 2010). However, HvNAC005 may be a relatively weak transcriptional activator since no activity was detectable under conditions which have previously been used to demonstrate activity for several NAC transcription factors (Jensen *et al.*, 2010; Kjaersgaard *et al.*, 2011). Members of the subgroup NAC-a6 closely related to HvNAC005 share four MEME sequence motifs in their disordered C-terminal regulatory domains (Fig. 2). Some of these motifs are likely to mediate protein–protein interactions of relevance to senescence. It is striking that AtNAP, a key senescence regulator (Guo and Gan, 2006), has a relatively short C-terminal regulatory domain and contains only one of the four conserved motifs. This motif, named the LL motif due to the significant conservation of two neighbouring leucine residues, is likely to be of importance for interactions with components of the basic transcription regulatory apparatus. Thus, deletion of the region containing the LL motif in HvNAC005 abolished the ability to activate transcription in yeast (Fig. 3). High divergence of subgroup-specific short C-terminal motifs (Ko *et al.*, 2007; Kjaersgaard *et al.*, 2011; Jensen and Skriver, 2014) demonstrates the molecular and functional complexity of the regulatory domains of NAC transcription factors.

Previous studies have claimed that over-expression of the NAP-like genes could lead to drought and/or abiotic stress tolerance, e.g. *TaNAC69*: Xue *et al.* (2011) and *OsNAP*: Chen *et al.* (2014). We did, in fact, make preliminary drought experiments in the greenhouse; however, these did not indicate any significant effects of *HvNAC005* over-expression on drought tolerance (data not shown). The strong phenotype of the over-expressing lines in our case may have obscured any positive effects that might be evident with plants with less severe phenotypes, as was seen in the studies of Chen *et al.* (2014).

Altogether, our studies demonstrate that, although the phylogenetically clustered NAC-a5/-a6 transcription factors share roles in the regulation of the senescence process, they also show differences in their structures, expression patterns, and functions, making characterization of individual NAC transcription factors relevant. Subtleties in their manner of regulation may be related to high diversification in both promoter regions of the genes and in the highly diverse C-terminal parts of the proteins. How this diversification translates into differences in regulation will be the aim of future investigations. Nonetheless, the current status points to NAC transcription factors as promising targets for future breeding to influence crop yields and nutrient use efficiency (Uauy *et al.*, 2006; Waters *et al.*, 2009), and fine-tuning the expression of NAC-a5/-a6 genes would be an obvious strategy due to their strong impact on development and senescence of the plants.

## Supplementary data

Supplementary data can be found at *JXB* online.

Figure S1. Alignment of promoter sequences for HvNAC005 and four closely related genes.

Figure S2. *HvNAC005* expression levels in 12 *HvNAC005* over-expression T_0_ lines.

Figure S3. Root characterization of T_1_ plants from *HvNAC005* over-expression line HvNAC005-OE-26.

Figure S4. Promoter sequences of two up-regulated genes, encoding a ZIM domain protein and a putative ZOG.

Table S1. Sequences of primers for cloning and check of transgenic lines.

Table S2. Accession numbers of HvNAC005-related proteins/genes.

Table S3. Genes, accession numbers, and primer sequences for qRT-PCR experiments.

Table S4. Mean number of days after sowing when half of the final number of spikes with visible awns had appeared.

Table S5. Mean number of tillers in plants of transgenic and control lines when the experiment was terminated.

Table S6. Distribution of differentially expressed genes in HvNAC005 over-expressing plants on functional MapMan BINs.

Supplementary Data
