# Supplementary material for: Barley plants over-expressing the NAC transcription factor gene HvNAC005 show stunting and delay in development combined with early senescence
Source: J Exp Bot. 2016 Jul 19;67(17):5259–73. doi: 10.1093/jxb/erw286 (PMC5014165; doi:10.1093/jxb/erw286)
Supplement: Supplementary Data [file supp_67_17_5259__index.html]

Barley plants over-expressing the NAC transcription factor gene HvNAC005 show stunting and delay in development combined with early senescence — Barley plants over-expressing the NAC transcription factor gene HvNAC005 show stunting and delay in development combined with early senescence — Supplementary Data 

# Barley plants over-expressing the NAC transcription factor gene *HvNAC005* show stunting and delay in development combined with early senescence

## Supplementary Data

Data files

- supplementary\_tables\_S1\_S5\_figures\_S1\_S4.pdf - Supplementary Data
- supplementary\_table\_S6.xlsx - Supplementary Data
