# Supplementary material for: Barley plants over-expressing the NAC transcription factor gene HvNAC005 show stunting and delay in development combined with early senescence
Source: J Exp Bot. 2016 Jul 19;67(17):5259–73. doi: 10.1093/jxb/erw286 (PMC5014165; doi:10.1093/jxb/erw286)
Supplement: Supplementary Data [file supp_erw286_supplementary_tables_S1_S5_figures_S1_S4.pdf]

Barley plants overexpressing the NAC transcription factor gene *HvNAC005* show stunting and delay in development combined with early senescence.

MW Christiansen, C Matthewman, D Podzimska-Sroka, C O´Shea. S Lindemose, NE Møllegaard, IB Holme, K Hebelstrup, K Skriver, PL Gregersen

Table S1. Sequences of specific primers used for cloning, for check of transgenic barley plants, and generation of different constructs.

**HvNAC005 cds PCR amplification – USER cloning**	
HvNAC005-Fw	5´-ATGATCATGTCCGACCCGG-3´
HvNAC005-Rev	5´-GATCGGTACCTTTAGAAATGGAGCAGCGGCT-3´
**Check of transgenic barley lines with qRT-PCR**	
NAC5-prom_junc_Fw	5´-ACAACCAGATCTCCCCCAAA-3´
NAC5-prom_junc_Rev	5´-TTGCGGAGGTAGTGCAGGAT-3´
NAC5-term_junc_Fw	5´-CCAAGTCGGCAACAACACCTTG-3´
NAC5-term_junc_Rev	5´-ATCATCGCAAGACCGGCAACAG-3´
**Genome walking for promoter cloning**	
Race-AP1:	5´-GTAATACGACTCACTATAGGGCACGCGTGG-3´
AP2:	5´-GGCACGCGTGGTCGACGGC-3´
HvNAC005-O:	5´-AACGCTCGAGGACGAAAACCCTAGCTG-3´
HvNAC005-I:	5´-AGCTGGCTAGCTAGAGCTTGGAGTGCT-3´
**Constructs for transactivation analysis in yeast**	
HvNAC005-FL-Fw	5´-ATATATCATATGATCATGTCCGACCCG-3´
HvNAC005-C-term-Fw	5´-GGAATTCCATATGAGCCAAGTGTCCCCGATGGCG-3´
HvNAC005-355-Rev	5´-CCGGAATTCTTACACCGCGGCCTGCAGTGT-3´
HvNAC005-319-Rev	5´-CCGGAATTCTTAGAGCGCGTGTCCGCTGTC-3´
HvNAC005-250-Rev	5´-CCGGAATTCTTACGCTGGCTGGCTCGTCGA-3´
HvNAC005-218-Rev	5´-CCGGAATTCTTAGAAATGGAGCAGCGGCTG-3´
**Promoter cloning for EMSA analysis**	
MLOC_61774 Fw	5´-GCTACGGGATCCCAACACCTCTCTTCCAATTTGA-3´
MLOC_61774 Rev	5´-CGTAGCTCTAGAGAAGCCGGTTGATGGCTTTGCT-3´
MLOC_61801 Fw	5´-GCTACGGGATCCACTCCAATAAAACAAATCTCACA-3´
MLOC_61801 Rev	5´-CGTAGCTCTAGAGGCAAGCATAGTGAGTGAACCT-3´

Table S2. Accession numbers of HvNAC005 related proteins/genes used for the phylogenetic tree in Fig. 2.

Name	Protein/gene acc. #	Database
ANAC018	AT1G52880.1	TAIR
ANAC025	AT1G61110.1	TAIR
ANAC047	AT3G04070.1	TAIR
ANAC056	AT3G15510.1	TAIR
ANAC060	AT3G44290.1	TAIR
AtNAP	AT1G69490.1	TAIR
BdNAC005	XP_003559782.1	GenBank
BdNAC006	XP_003562891.1	GenBank
BdNAC023	XP_003557998.1	GenBank
BdNAC090	XP_003577115.1	GenBank
BeNAC1	HM626401.1	GenBank
BnNAC2	AFI56994.1	GenBank
GmNAC26	NP_001238204.1	GenBank
HvNAC005	AK251058.1	GenBank
HvNAC023	CBZ41159.1	GenBank
HvNAC027	BAJ99416.1	GenBank
HvNAC030	ABI94358.1	GenBank
ONAC010	LOC_Os07g37920.1	MSU/RAP-DB
ONAC016	LOC_Os01g01430.1	MSU/RAP-DB
ONAC058	LOC_Os03g21060.1	MSU/RAP-DB
ONAC103	LOC_Os07g48450.1	MSU/RAP-DB
ONAC122	LOC_Os11g03300.1	MSU/RAP-DB
ONAC131	LOC_Os12g03040.1	MSU/RAP-DB
StNAC2	NP_001275015.1	GenBank
TaNAC005-like	W5EN00_WHEAT	uniprot_trembl
TaNAC25	ADG85701.1	GenBank
TaNAC69	AAU08785.1	GenBank
TtNAM-A1	A0SPJ3.1	GenBank
TtNAM-B1	A0SPJ4.1	GenBank
TtNAM-B2	A0SPJ6.1	GenBank
ZmNAC118	GRMZM2G109627_T01	MaizeGDB
ZmNAC122	GRMZM2G430849_T01	MaizeGDB
ZmNAC25	GRMZM2G127379_T01	MaizeGDB
ZmNAC44	GRMZM2G011598_T01	MaizeGDB
ZmNAC81	GRMZM2G042494	MaizeGDB

Table S3: Genes, accession numbers (GenBank), and primer sequences for qRT-PCR gene expression experiments performed in this work.

Gene	Accession	Fw-primer	Rv-primer
18S ribosomal RNA	AY552749.1	ACGGCTACCACATCCAAGGA	CAGGATTGGGTAATTTGCGC
9-cis-epoxycarotenoid dioxygenase (NCED1)	AK365103.1	CGCCCTCCATCCCTCCCATCTTCT	CCGCCGCTAACTGTTTCCTCTTCC
Amino acid permease	AK361903.1	ACTTCGGCCCAGAAGATATGGC	AGTAGGCGAATGCGATGTTCCC
APETALA2 and ethylene responsive element binding protein	AK374826.1	TGATGGACGACGGCAACTTCTG	ATGTTGTGATGGGCGGTGATCC
CYSPEP	AM941122.1	AAAGGTGGCAAGGATTATTGG	TGCCACAGATACCTGACGAT
HvNAC005	AK251058.1	CCATGTGAACAGCAGCGGCAAC	CCGACGTTGAGGCTGGTGAATC
HvNAC013	AK376297.1	ATGCCGCCGCACATGATGTAC	ACAGGTCGCCGGAATTAGCG
HvNAC027	FR819765.1	ACGGCTACGTGAACCACGACAC	CAAGCTGCCGCTGGATCTCTTC
Jasmonate ZIM domain-containing protein	AK358513.1	CAGCAGAGCAACAAGCTCAACG	TGGGCTGGTGAGTAAGTATCTGC
Nuclease I	AB028448.1	ACTATTACTTCCGGGCGTTGCC	TCTGCAGCCTGCTATTCTTCCC
Oxoglutarate/iron dependent dioxygenase	AK355337.1	CAGCAGAGCAACAAGCTCAACG	TGGGCTGGTGAGTAAGTATCTGC
Pheophorbide a oxygenase (PaO)	AK358479.1	AGGCATCGAGAACTGGCAGAAG	ACCAGTTCCTGAATGCGACGAC
Protein phosphatase 2C	BM816007	TCGCAATGTCACGGTCAATCGG	ACGCTGACTTCAGGCTTTGGAATC
Protein phosphatase 2C	AK367469.1	TGCTCACCAAGATGGCTCTCAC	GAGATCGACGACGACAACAGAG
Protein phosphatase 2C	AK374059.1	TCTTCCTTCCGTTCCCTCGTTC	CAACAGCTGAGCTTCTCCGTTC
RUBISCO small subunit	AB020943.1	TCTTCCGTGAGCACAACAGC	TCCAGTATCGGCCGTCGTAG
Saccharopine dehydrogenase	AJ508229.2	GTTCAGAGGAAAGGCGTGATCC	ACTCTCTCGATCAGCTGAATGCC
Stay green protein (SGR)	AY850135.1	CGTGGTTGTTGTACGGGCAAAG	CAGTGGGATCTTGTTGCAGCATCG
Splicing factor 2 (SP2)	AK249101.1	GAAGGATGAGTAGGCGCTGG	CTGGGAGGTTCCCAACGTAA
Zeatin O-glucosyl-transferase (ZOG)	AK364192.1	AGAAGAAGGCACACGAGTGGAG	TCGTCGAAGTTGCGGTGAGATG

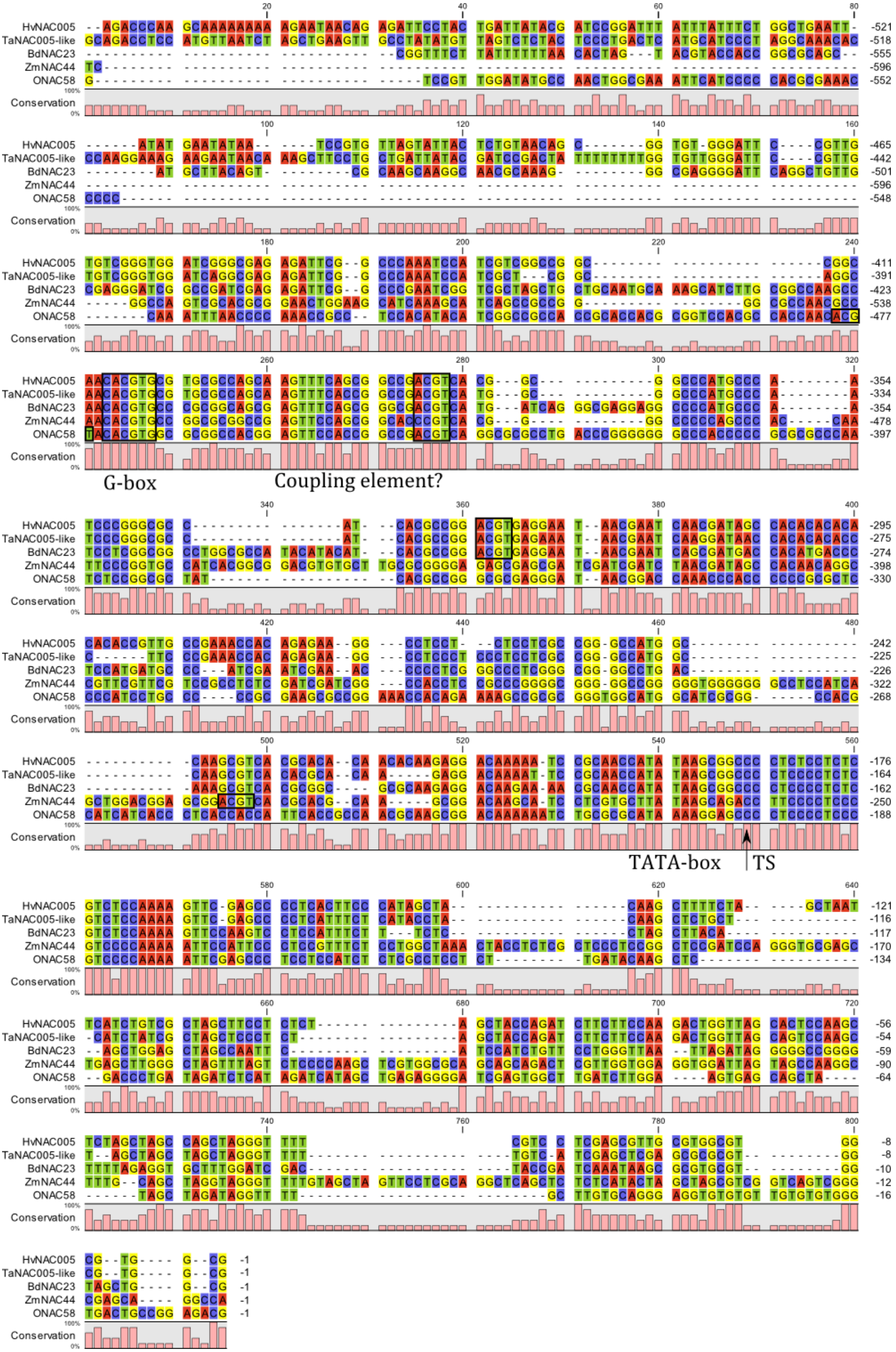
HvNAC005
TaNAC005-like
BdNAC23
ZmNAC44
ONAC58
Conservation
G-box
Coupling element?
TATA-box
TS

Fig S1. Alignment of the promoter (600 bp upstream of ATG) of HvNAC005 and the four most closely related genes from wheat, Brachypodium, maize and rice (see Fig. 2). G-box: an ABRE-like CACGTG motif. TS: transcription start. A potential ABRE coupling element is indicated, as well as the putative TATA box. ACGT containing motifs are delineated by black rectangles.

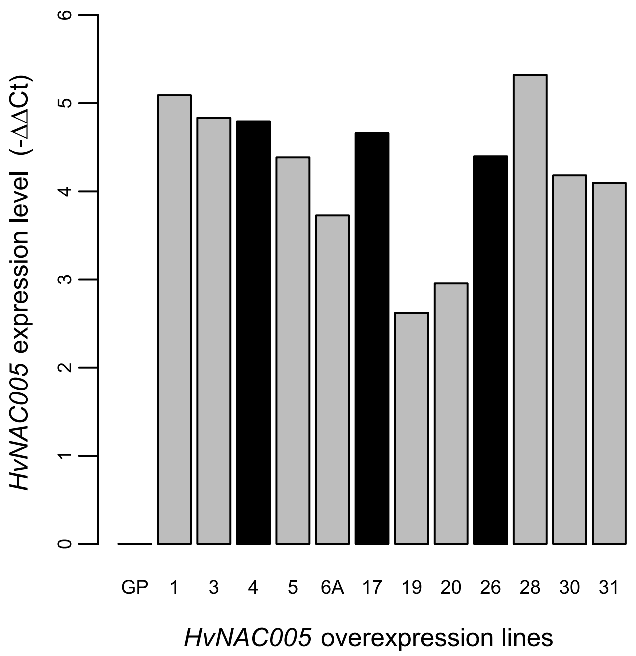

Fig. S2. *HvNAC005* expression level, as determined by qRT-PCR, for 12 *HvNAC005* overexpression T_0 lines. Gene expression levels (-ΔΔCt, i.e. log2 scale) were normalized to the expression level of *18S rRNA* and with barley 'Golden Promise' (GP) control line as the calibrator sample. Since only one plant per line was available, the determinations are based on only one biological replicate with three technical replicates, except for GP and line 26 (T_1 plants), which are based on 4 biological replicates. Black bars: the three lines that were used for further characterization of *HvNAC005* overexpressing plants. Gray bars: lines that were not used for further characterization, due to bad seed setting or low expression of *HvNAC005*.

Table S4. Mean number of days after sowing when half of the final number of spikes with visible awns had appeared. Numbers of awns were recorded twice a week, when SPAD measurements were taken, placing the number of days into slots with 3-5 days intervals. A t-test was used to estimate statistical delays in this number in HvNAC005 over-expression lines.

Line	Number of plants	Days after sowing +/- SE	P-value, t-test
HvNAC005-OE-4	4	97.0 +/- 1.1	0.028*
HvNAC005-OE-4-null	6	93.0 +/- 1.0	
HvNAC005-OE-17	3	100.7 +/-2.3	0.191^{NS}
HvNAC005-OE-17-null	3	96.3 +/- 0.75	
HvNAC005-OE-26	4	98.2 +/- 0.75	0.004**
HvNAC005-OE-26-null	5	94.0 +/- 0.0	
Golden Promise	4	95.5 +/- 0.87	
null vs. Golden Promise		94.4 vs. 95.5	0.335^{NS}
OE vs. Golden Promise		98.2 vs. 95.5	0.045*

Table S5. Mean number of tillers in plants of transgenic and control lines when the experiment was terminated 116 days after sowing. A t-test was used to estimate statistical differences among the lines.

Line	Number of plants	Final number of tillers +/- SE	P-value, t-test
HvNAC005-OE-4	5	96 +/- 13	0.19^{NS}
HvNAC005-OE-4- null	4	79 +/- 8	
HvNAC005-OE-17	3	130 +/- 27	0.57^{NS}
HvNAC005-OE-17- null	3	111 +/- 10	
HvNAC005-OE-26	3	100 +/- 3	0.049*
HvNAC005-OE-26- null	4	119 +/- 7	
Golden Promise	4	92 +/- 11	

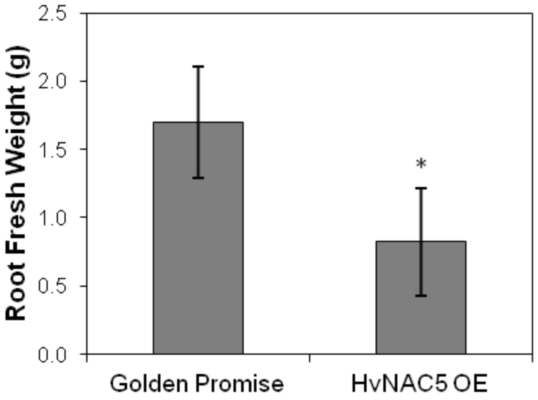

Fig. S3. Characterization of T_1 plants from *HvNAC005* overexpression line HvNAC005-OE-26. Fresh weight of roots from four week old seedlings. Error bars: standard deviation. Asterisk indicate reduced root mass in the HvNAC005-OE line, $p<0.05$ (t-test).

A) MLOC_61774|MLOC_61774.1

CAACACCTCTCTTCCAATTTGAGCTTCAAGTACAACCACAAATGGAGTGGCATCTTCAAA
CCGAGCACCATTCCCTTCACCTCTTAGCCTACACACTTCATCTCACTTATCTCCAATCAC
ATCAGTCAAAAACTTCGGCAATGGATCATCATGCCATTCAATAAATCCACAACCACCATG
TTGTTCAATTTTAGTAATAGAAATCAATCAGTGGGGAGGAGAACAAATGAAGAGAAACAA
ACAGAAATCCAACTCACCATGGCATCGATGCATGCGTAGTACCTTCTGCCTGGATTATGA
ATGCTCGAGGAGATCCATCTTGACGCCTTCCGACGAGGATTGCACCAGCAAAGCTTCTGC
GGCTCGAACGCCATCGGGCTCTCCCTATAAAGCACCGATGACCTCGACTCCCCCCTCTCC
TTCCTGCGGCTCAACGAAAGCCAGGAGCAGCAGAATGACCCGATGACCACGACATTCCAT
TGGACAGATCCGCCGCCGCCGCCCCTCTCCCTAGTTCTCCCACTCGAAAATCCCCAATTA
ATTTGATGTTCCCTCATCTCTCCCCGCAACGCGGTTATAATGCAACCTGGCCCATTAGTC
ATAAAGAATTCCGGTAGACTCTTGCCATGTCATCATGTTTAGCTCTAAATGACGTTAGGT
TTTAATTTAGACCGGAATTTATAAATTTAGAGCATAAACAATAAGGATTTTAACTTCAGA
GTTCGGTTCGCTCAAGGACTAGAGTTCAATGTTTAAATGGACCTTTTCCGTTTGTGAGCT
CTGTGTCTGCCGCCGAGTTAACAACTGAACGTGCGGCACGAGCGGAGTGCGAGCCACCAC
GTCGAAAATGTCTTGCTGTCCCAGTAATCATTGTATAATCTCCGTCGACCAGCACGTGTT
CTTCGTGCGAGAGACGTCCAGGAAGAAACACCCACATTAGACAGCAACAGCCCACAGGAC
ACGCTTCGATTCCTATAAAGCAAAGCCATCAACCGGCTTC

B) MLOC_61801|MLOC_61801.1

ACTCCAATAAAACAAATCTCACATTCACCTAGCTCCACGACCTGTCGCATACGTTGACAC
CCCTTTGCTAGAATCCTCATCCTCATCGCCGGCATCCTTAATGTCATCCACACTAGGGAG
AACATGAAGGCAACCAAGAAGACACACATGGAGGGAACTGAGACAGGTGTCGCAACTAGG
AGTCCAAGCAAACATTTCAATACAATAGCCAAAGGAAATGGCCTTGAGAATAGCAGCTTG
TTTGCTGGCACCGTTAGTGGTAGCAAACAAAACGGTGATGATATTCTCAAGGTTGTTGTC
CTTGAAGACTACAACGGCTTTCTTCTTTGCACCCGTAGCGACACTTGTCACAGGTCCAAT
CCTTGTGCTTCTTCTTGGTCGCGTGAGCATGGCCACATAATTTGGATATTTATCATTTGC
GGCGCTCAAACTCCGGATGACCATTCAACTCTTCTTTGCATGTGGCGATGCAGATCTCCC
AATCTACGGATAATGCTCGTGTGTCTAGTTTGATCTTGATGATTAATTGTGCTAACATAA
TTTTCATGGTTCTTTTTTGGATTAATCTAATTTGAAAATTAATATTTTTATTTTAACACA
GTGTGATTTGGACAGGACAAGGAGCATCGGATGATGGTTCCACGTAGGGACTTAGGAGAG
AAAACTACGACCGGCCATTTTGGGGAAGACTCGAGCAAGGCTGCCATGTAAAAACTTCTA
TTCCTTCTGTATCTAAATAATTATATTTGAAAAAAACTAAAGTAAAAGAGATTATATTTA
GAAACGGAGCAAGTAGCAATTGAAGCGAGTAATAGATCCACCCGACATGTCCATGTGGAG
CTTAATTCCACTGAAAGTTTTCATCCTCGATCCAAGGTTAATTATATGAACTACAATAAA
GAACCAATACGTCCCAATCGGTCGACGTAATCACTTGCGTACGTCCATCATACCACGACA
CTATTTGAGAGACGTCCTAGGTTCACTCACTATGCTTGCC

Fig. S4. Promoter sequences of two genes selected among the upregulated genes in the gene expression microarray for *HvNAC005* overexpressing plants. CGT/ACG (yellow/green) motifs potentially contained in NACBSs are highlighted. A) Gene encoding a ZIM domain protein (EnsemblPlants: MLOC_61774.1; GenBank: AK358513.1). B) Gene encoding a putative zeatin glucosyltransferase (ZOG) (EnsemblPlants: MLOC_61801.1; GenBank: AK364192.1).
